# FER-1/Dysferlin promotes cholinergic signaling at the neuromuscular junction in *C. elegans* and mice

**DOI:** 10.1242/bio.20135637

**Published:** 2013-10-15

**Authors:** Predrag Krajacic, Emidio E. Pistilli, Jessica E. Tanis, Tejvir S. Khurana, S. Todd Lamitina

**Affiliations:** 1 Department of Physiology, Richards Research Building A702, University of Pennsylvania, Philadelphia, PA 19104, USA; 2 Pennsylvania Muscle Institute, 700A Clinical Research Building, University of Pennsylvania, Philadelphia, PA 19104, USA; ‡ Present address: West Virginia University, Center for Cardiovascular and Respiratory Sciences, PO Box 9105, Morgantown, WV 26506, USA

**Keywords:** Dysferlin, Muscular dystrophy, LGMD2B, Limb-girdle, Synaptic transmission

## Abstract

Dysferlin is a member of the evolutionarily conserved ferlin gene family. Mutations in Dysferlin lead to Limb Girdle Muscular Dystrophy 2B (LGMD2B), an inherited, progressive and incurable muscle disorder. However, the molecular mechanisms underlying disease pathogenesis are not fully understood. We found that both loss-of-function mutations and muscle-specific overexpression of *C. elegans fer-1*, the founding member of the Dysferlin gene family, caused defects in muscle cholinergic signaling. To determine if Dysferlin-dependent regulation of cholinergic signaling is evolutionarily conserved, we examined the *in vivo* physiological properties of skeletal muscle synaptic signaling in a mouse model of Dysferlin-deficiency. In addition to a loss in muscle strength, Dysferlin −/− mice also exhibited a cholinergic deficit manifested by a progressive, frequency-dependent decrement in their compound muscle action potentials following repetitive nerve stimulation, which was observed in another Dysferlin mouse model but not in a Dysferlin-independent mouse model of muscular dystrophy. Oral administration of Pyridostigmine bromide, a clinically used acetylcholinesterase inhibitor (AchE.I) known to increase synaptic efficacy, reversed the action potential defect and restored *in vivo* muscle strength to Dysferlin −/− mice without altering muscle pathophysiology. Our data demonstrate a previously unappreciated role for Dysferlin in the regulation of cholinergic signaling and suggest that such regulation may play a significant pathophysiological role in LGMD2B disease.

## Introduction

Limb-Girdle Muscular Dystrophy 2B, or Dysferlinopathy, is an incurable muscle disorder in which patients usually present in the second or third decade of life with proximal and/or distal muscle weakness, elevated serum creatine kinase (CK) levels, and generally slow disease progression (Amato and Brown, 2011). Both LGMD2B and a related disorder, Miyoshi Myopathy (MM) are caused by loss-of-function mutations in the Dysferlin gene product (Amato and Brown, 2011). Analysis of muscles lacking Dysferlin function reveals a sub-sarcolemmal accumulation of membrane vesicles, suggesting defects in vesicle turnover (Bansal et al., 2003; Ho et al., 2004). Additionally, the loss of Dysferlin leads to immunity defects, such as increased levels of phagocytic macrophages and susceptibility to complement attack (Wenzel et al., 2005; Nagaraju et al., 2008). Recent studies demonstrate that restoration of Dyferlin gene expression solely in the skeletal muscle is sufficient to rescue all disease phenotypes (Millay et al., 2009), suggesting that the immune functions of Dysferlin play minor roles in disease pathogenesis and that the pathophysiological defect(s) associated with the loss of Dysferlin is based within the muscle. Defining these muscle-specific roles of Dysferlin could provide insight into LGMD2B pathogenesis and may suggest therapeutic opportunities for this untreatable disease.

Dysferlin is part of the ferlin-1 like protein family, which also include otoferlin (Fer1L2) and myoferlin (Fer1L3), Fer1L4, Fer1L5, and Fer1L6 (Han and Campbell, 2007). Like the other ferlins, Dysferlin encodes a large (230 kD) protein with multiple calcium and phospholipid-binding C2 domains and a carboxy-terminal transmembrane domain. Dysferlin expression is highly enriched in skeletal muscle and is also present in other tissues, including brain and heart (Bashir et al., 1998; Liu et al., 1998). In skeletal muscle, Dysferlin is thought to promote damage-induced membrane repair in a Ca^2+^ dependent manner and Dysferlin mutants are deficient in this process (Bansal et al., 2003). Until recently, the current model for pathogenesis of LGMD2B suggested that mutations in Dysferlin inhibit the active repair of muscle membranes, resulting in the progressive accumulation of damaged muscle fibers and eventual manifestation of the dystrophic phenotype (Han and Campbell, 2007). However, a new study suggests that defective membrane repair may not be the cause of the disease (Lostal et al., 2012). Whether Dysferlin regulates other aspects of muscle physiology and whether these processes play a significant role in disease pathogenesis has not been determined.

The *C. elegans* gene *fer-1* encodes the founding member of the ferlin gene family (Bashir et al., 1998; Liu et al., 1998) and is homologous to all six human ferlin-1-like proteins (Lek et al., 2010). Loss-of-function *fer-1* mutants are sterile due to defects in spermatogenesis. While the original characterization of *C. elegans fer-1* noted expression outside of sperm (Achanzar and Ward, 1997) and we recently demonstrated expression of *fer-1* mRNA in *C. elegans* muscle (Krajacic et al., 2009), a functional role for *fer-1* in *C. elegans* muscle has not been explored. Here, we present evidence that, in addition to its well described role in spermatogenesis, *fer-1* is also expressed in the *C. elegans* body wall muscle cells, which are the functional and anatomical equivalent of mammalian skeletal muscles. Surprisingly, we find that multiple *fer-1* mutants, as well as animals overexpressing *fer-1* specifically in muscle, exhibit defects in muscle cholinergic signaling, a function not previously ascribed to Dysferlin in any system. We also show that mice carrying loss-of-function mutations in Dysferlin exhibit defects in muscle cholinergic signaling, suggesting that the synaptic function of Dysferlin is evolutionarily conserved. Treatment of Dysferlin mutant mice with the FDA-approved acetylcholinesterase inhibitor (AchE.I) Pyridostigmine bromide reversed Dysferlin-dependent cholinergic defects and restored muscle strength. Our findings suggest that decreased efficiency of post-synaptic cholinergic signaling may also contribute to the pathogenesis of LGMD2B and could represent a novel target for disease therapies.

## Results

**Fig. 1.**
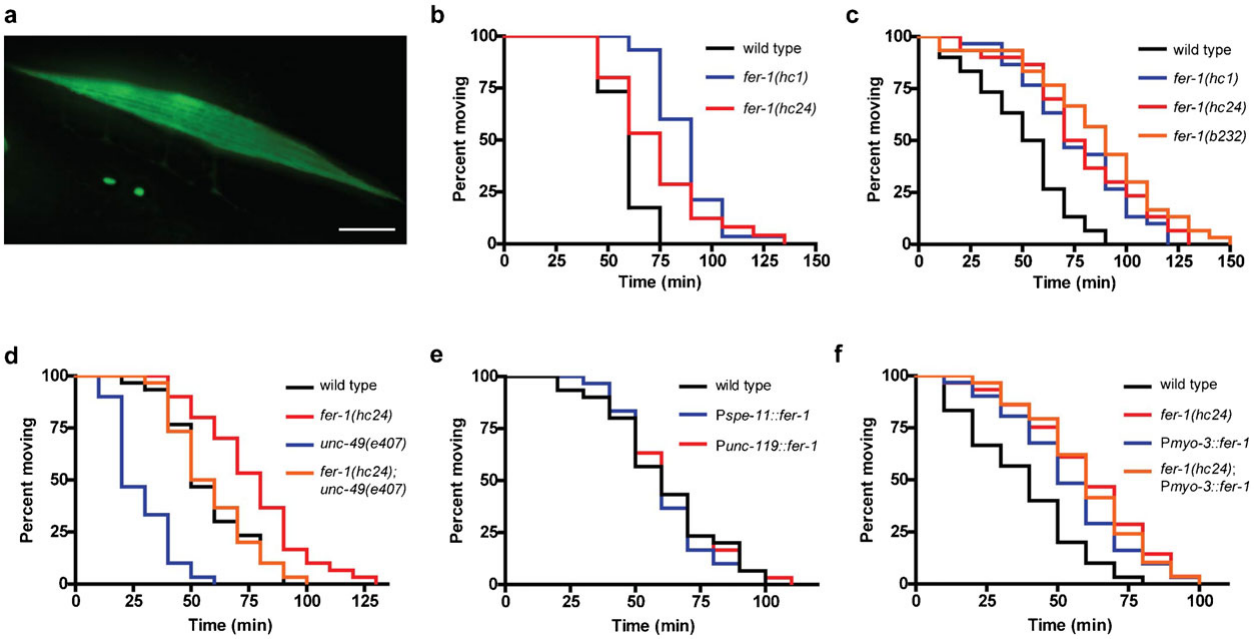
*fer-1* mutants exhibit defects in cholinergic synaptic function. (a) GFP fluorescence in animals carrying a *fer-1* promoter::*gfp*::*unc-54* 3′ UTR reporter transgene shows expression in the body-wall muscles. Scale bar = 20 µm. (b) Loss of *fer-1* causes resistance to paralysis induced by the cholinesterase inhibitor aldicarb; *P*<0.005. (c) *fer-1* mutants exhibit increased resistance to the paralytic effects of the L-AchR agonist levamisole; *P*<0.001. (d) Loss of GABA signaling in the *unc-49* mutant causes hypersensitivity to levamisole (*P*<0.0001) that is suppressed by the loss of *fer-1* (*P*<0.0001). (e) Overexpression of *fer-1* in either the sperm or neurons is not sufficient to alter levamisole sensitivity; *P* = 0.90. (f) Overexpression of *fer-1* in the body-wall muscles causes levamisole resistance; *P*<0.005. For (b–f), each Kaplan–Meyer graph shows data from one representative experiment, *n* = 30 animals/genotype.

To gain more insights into the role of Dysferlin, we re-examined the function of the founding member of the Dysferlin gene family, *C. elegans fer-1*. In addition to its previously well described expression in sperm (Achanzar and Ward, 1997), we also found that a *fer-1* promoter::*gfp* reporter showed expression in body wall muscle cells (Fig. 1a), consistent with previous mRNA expression analysis of purified muscle cells (Krajacic et al., 2009). *fer-1* mutants exhibit normal sarcomere structure (Krajacic et al., 2009) and proper muscle morphology (supplementary material Fig. S1), suggesting that *fer-1* is not required for muscle development or for the maintenance of muscle sarcomeres.

Although *fer-1* mutants do not exhibit signs of muscle damage, we considered the possibility that they might show defects in muscle functional properties. Given that other ferlin family members are known to regulate synaptic transmission (Roux et al., 2006), we explored whether *fer-1* might also play a role in signaling at the neuromuscular junction (NMJ). In *C. elegans*, NMJ signaling is reciprocally regulated by a single inhibitory GABA receptor and two distinct excitatory acetylcholine receptors (AchRs) (Richmond and Jorgensen, 1999). The balance between GABA and AchR signaling can be probed with the acetylcholinesterase inhibitor aldicarb and the AchR agonist levamisole. Both drugs cause excitation of post-synaptic AchRs, muscle hypercontraction, and time-dependent paralysis (Mahoney et al., 2006). In animals treated with aldicarb the time to paralysis is indicative of either the rate of pre-synaptic Ach/GABA release or post-synaptic Ach/GABA signaling. In contrast, the time to paralysis for animals treated with levamisole can be used to identify defects in post-synaptic signaling (Mahoney et al., 2006). We found that multiple *fer-1* loss of function mutants, including the putative null allele *hc47* (supplementary material Fig. S2) were weakly resistant to both aldicarb (Fig. 1b) and levamisole (Fig. 1c). As has been described for *fer-1* spermatogenesis defective phenotypes, the levamisole resistance for all non-null *fer-1* alleles was also temperature-sensitive, suggesting that the genetic basis of the *fer-1* synaptic phenotype is loss-of-function, as has been previously demonstrated for *fer-1* sperm phenotypes (Ward and Miwa, 1978). Along with our previous observations demonstrating *fer-1* mRNA expression in purified cultured muscle cells but not in purified neuronal cells (Krajacic et al., 2009), these findings suggest that FER-1 acts in post-synaptic body wall muscle cells (Mahoney et al., 2006). Resistance to cholinergic stimulation was also observed in *fer-1* mutants lacking a germline, but not in another related fertility mutant, *spe-5*, demonstrating that resistance was due to *fer-1* somatic functions and not to *fer-1* sterility (supplementary material Fig. S3).

Levamisole resistance could result from either enhanced inhibitory signaling (via GABA receptors) or reduced excitatory signaling (via Ach receptors) at the NMJ (Richmond and Jorgensen, 1999). To distinguish between these possibilities, we examined the levamisole resistance of *fer-1* mutants in an *unc-49* mutant background, which encodes the sole *C. elegans* ionotropic GABA receptor. Compared to the *unc-49* single mutant, levamisole resistance was still observed in the *fer-1;unc-49* double mutant (Fig. 1d), suggesting that loss of *fer-1* does not cause levamisole resistance through enhanced GABA signaling. The mechanism by which loss of *fer-1* disrupted cholinergic signaling did not appear to involve the steady-state clustering of L-AchRs since the localization of an UNC-63::YFP fusion protein (Gendrel et al., 2009) appeared normal in multiple *fer-1* mutants (supplementary material Fig. S2). Together, these data suggest that *fer-1* reduces cholinergic signaling at the *C. elegans* NMJ.

In mice, muscle-specific overexpression of Dysferlin gives rise to a progressive muscular dystrophy, although this phenotype is distinct from that caused by loss of Dysferlin since there is no evidence of sarcolemmal membrane damage (Glover et al., 2010). Given that *fer-1* is expressed in *C. elegans* muscle, we hypothesized that muscle-specific overexpression of *fer-1* might also cause muscle defects, possibly by disrupting AchR signaling. To test this hypothesis, we overexpressed the *fer-1* genomic coding sequence under the control of the muscle specific *myo-3* promoter, the neuron-specific *unc-119* promoter, or the sperm-specific *spe-11* promoter using the single-copy insertion method (Frøkjær-Jensen et al., 2008). The *fer-1* single-copy expression clone was functional since the *spe-11p::fer-1* transgene was able to rescue the sterility of *fer-1(hc47)* animals (data not shown). Animals overexpressing *fer-1* in either the sperm or the neurons exhibited normal levamisole response (Fig. 1e). However, animals overexpressing *fer-1* in the muscle exhibited levamisole resistance equivalent to that seen in *fer-1* loss-of-function mutants (Fig. 1f). Introduction of the *myo-3p::fer-1* transgene into the *fer-1(hc24)* background did not rescue or enhance *fer-1* mutant levamisole resistance (Fig. 1f), suggesting that *fer-1* overexpression disrupts a similar pathway as that affected by *fer-1* loss-of-function. These data suggest that overexpression of *fer-1* in *C. elegans* muscle, but not in neurons or sperm, can phenocopy *fer-1* loss-of-function mutants and reduce cholinergic signaling in *C. elegans*. However, it remains possible that the mechanism by which *fer-1* overexpression reduces cholinergic signaling is distinct from that caused by loss of *fer-1*.

**Fig. 2.**
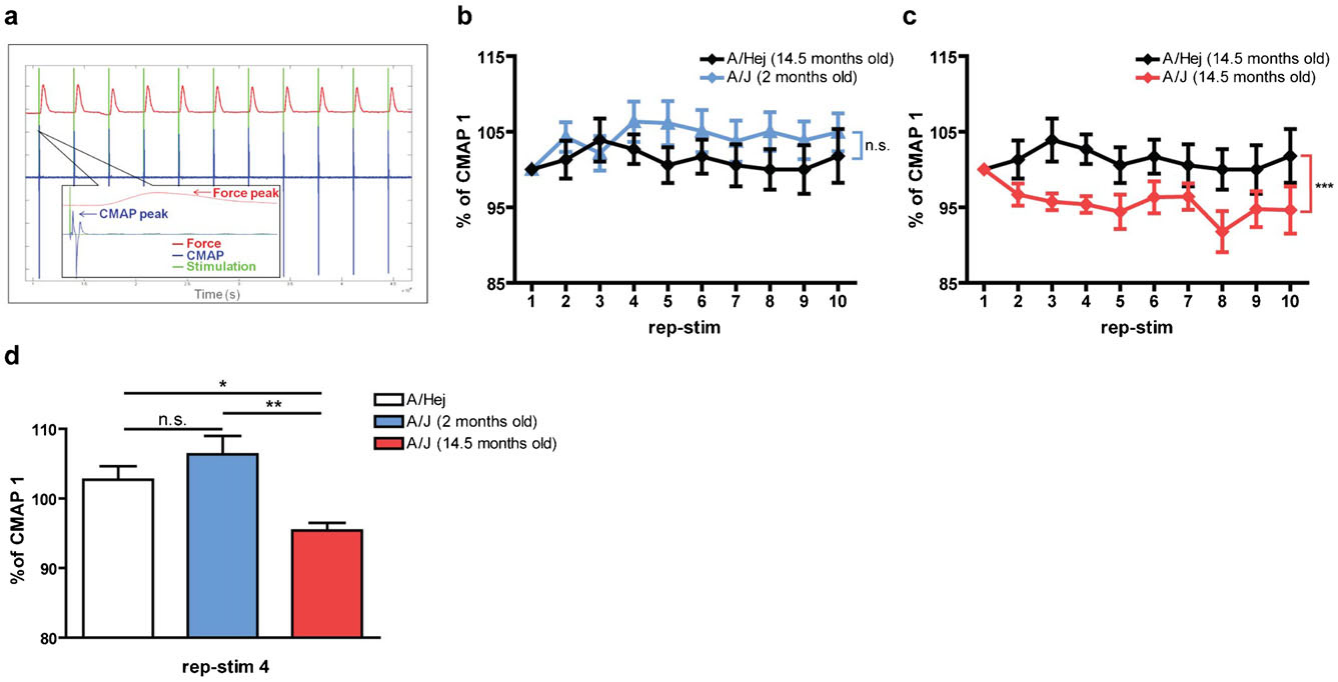
Dysferlin deficient A/J mice show age-dependent CMAP decrement upon repetitive stimulation. (a) Representative raw data traces of simultaneously recorded stimulation (green), CMAP (blue) and dorsiflexion force (red). Insert: one rep-stim cycle. (b,c) Normalized mean CMAP voltage on 3 Hz repetitive nerve stimulation, expressed as a % of CMAP 1, for 2 months A/J (*n* = 8) and 14.5 months old A/J mice (*n* = 7) compared to 14.5 months old A/HeJ mice (*n* = 7). (d) Normalized mean CMAP voltage on rep-stim 4. One way ANOVA with Bonferroni's multiple comparison test: *P<0.05; **P<0.01; ***P<0.001.

The effect of *fer-1* on *C. elegans* AchR signaling could be due to unique features of worm neuroanatomy/physiology or could represent a functionally significant, but previously undescribed role for Dysferlin. Therefore, we explored the effect of a Dysferlin loss-of-function mutation on cholinergic signaling at the mouse NMJ using the well-established A/J Dysferlin mutant mouse model (Ho et al., 2004; Wenzel et al., 2007; Millay et al., 2009). Muscle pathophysiology in A/J mice is progressive (observable histological muscle damage not present until ∼6 months of age, followed by relatively slow progression). To determine if loss of Dysferlin altered cholinergic signaling at the NMJ, we developed an *in vivo* physiological apparatus (supplementary material Fig. S4) that allowed us to perform repetitive nerve stimulation (RNS) of the peroneal nerve at a defined frequency while simultaneously monitoring the Ach-dependent compound muscle action potential (CMAP) through electromyography (EMG) and muscle force production with a force transducer (Fig. 2a). Aged wild type mice (15 month old A/HeJ) could undergo RNS at either 0.1 Hz or 3 Hz without becoming tetanic or exhibiting significant CMAP or force drops (Table 1). However, aged A/J mice, but not young A/J mice, exhibited a significant CMAP drop at RNS frequencies of 3.0 Hz that was not observed at 0.1 Hz (Fig. 2b,c; Table 1). A similar frequency-dependent CMAP drop was also observed in a different mouse Dysferlin mutant (SJL/J model), suggesting that this phenotype is due to loss of Dysferlin and not to other defects associated with a specific mouse strain (supplementary material Fig. S5). Furthermore, we did not observe these phenotypes in the unrelated mouse muscular dystrophy mutant (*mdx* dystrophin mutant, supplementary material Fig. S5) (Bittner et al., 1999; Turk et al., 2006). Given that *mdx* mice exhibit similar if not greater levels of muscle degeneration/regeneration than Dysferlin mutant mice, these data suggest that the cholinergic signaling defects in Dysferlin mutant mice are not due to generalized muscle damage or regeneration. They are also not simply due to aging since age-matched wild type controls do not exhibit similar cholinergic signaling defects.

**Table 1.**
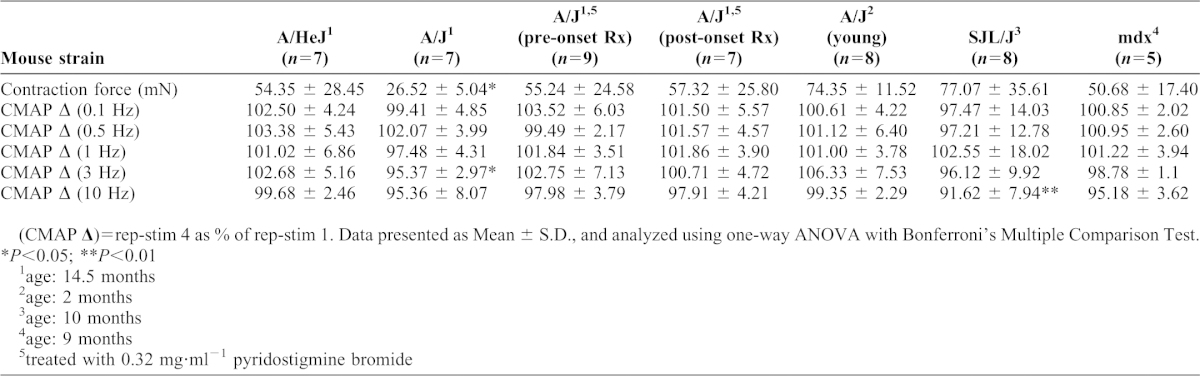
*In vivo* muscle physiology.

We also examined the *ex vivo* properties of electrically evoked twitch and tetanic contractions in EDL muscles of A/HeJ, A/J and SJL/J mice. For all genotypes and age groups except for SJL/J, the normalized *ex vivo* twitch and tetanic forces were not different between groups (supplementary material Table S1). A small but significant decrease in normalized twitch force, but not normalized tetanic force, was observed in SJL/J (supplementary material Table S1). Taken together, these data suggest that loss of Dysferlin has little, if any, effect on the contractile properties of muscle under *ex vivo* conditions that bypass the neuromuscular synapse.

**Fig. 3.**
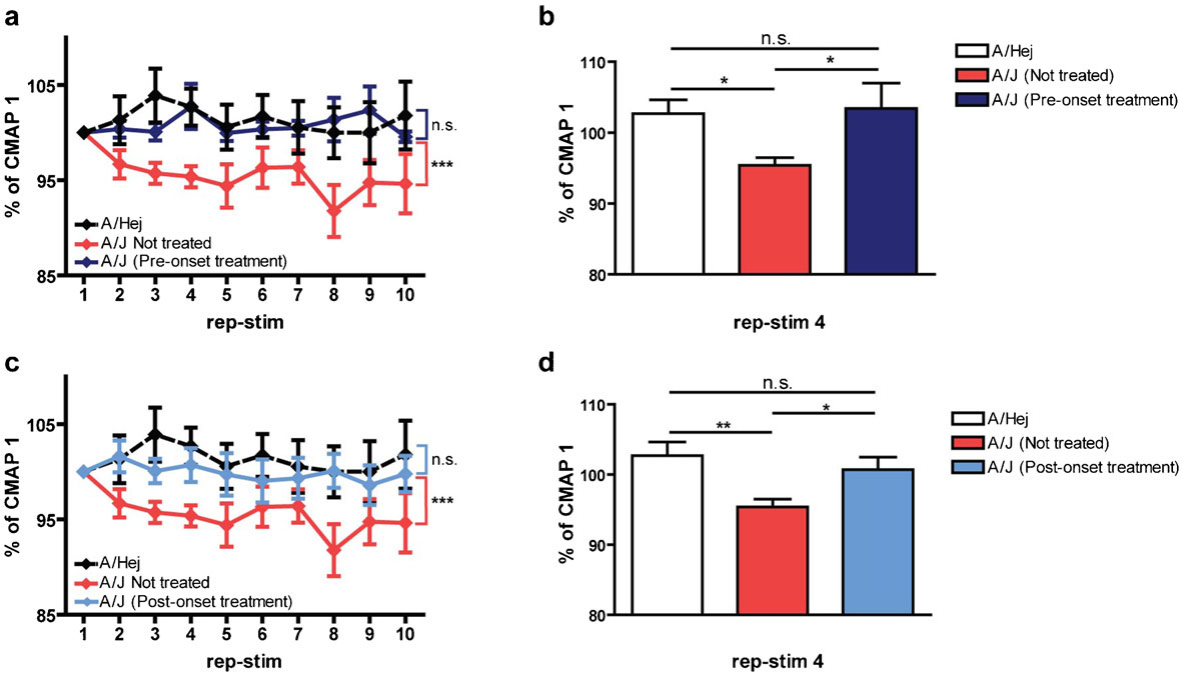
Dysferlin deficient A/J mice CMAP defect can be corrected with Acetylcholinesterase inhibitors. (a,c) Normalized mean CMAP voltage (3 Hz repetitive nerve stimulation) expressed as a % of CMAP 1. (a) CMAP decrement in A/J mice treated with Pyridostigmine bromide (A/J pre-onset group, *n* = 9). Treatment started before the disease onset at 2 months of age and continued until 14 months of age. Age matched control groups (A/HeJ, *n* = 7; A/J, *n* = 7) received no treatment. (b) Normalized mean CMAP voltage on rep-stim 4. (c) CMAP decrement in A/J mice treated with Pyridostigmine bromide (A/J post-onset group, *n* = 8). Treatment started after the disease onset at 7 months of age and continued until 14 months of age. Age matched control groups (A/HeJ, *n* = 7; A/J, *n* = 7) received no treatment. (d) Normalized CMAP voltage on rep-stim 4. 1 way ANOVA with Bonferroni's multiple comparison test: *P<0.05; **P<0.01; ***P<0.001.

The CMAP defect observed in Dysferlin mutants loosely resembles that found in Myasthenic syndromes, where a reduction in cholinergic signaling brought about through a variety of molecular mechanisms causes a RNS CMAP decrement, congenital fatigueability, and loss of muscle strength (Hirsch, 2007). While our findings above suggest a weak RNS CMAP decrement and loss of muscle strength associated with loss of Dysferlin, LGMD2B patients are not thought to exhibit fatigueability and are often sportive through childhood and early adult years, which indicates that LGMD2B is not a classical myasthenic syndrome. Still, we considered the possibility that loss of Dysferlin may alter cholinergic signaling in a way that resembles other bonafide cholinergic disease states. These syndromes are often responsive to small molecule inhibitors of AchE (AchE.I), the enzyme that breaks down Ach in the synaptic cleft of the NMJ. We hypothesized that if the CMAP defect caused by loss of Dysferlin was similar to that found in myasthenic conditions, then the Dysferlin CMAP defect might be improved via AchE.I therapy. Furthermore, if the CMAP defect is a primary cause of reduced muscle performance (rather than a secondary consequence of disease), then AchE.I therapy should also improve *in vivo* muscle strength in A/J mice. To test this hypothesis, we treated A/J mice with the AchE.I Pyridostigmine bromide starting either pre-pathologically (treatment beginning at 2 months of age) or co-pathologically (treatment beginning at 7 months of age) and then measured their *in vivo* CMAPs, muscle performance, and muscle histology. Both pre-pathological treatment (Fig. 3a,b) and co-pathological treatment (Fig. 3c,d) eliminated the CMAP drop found in Dysferlin mutants. Both AchE.I therapeutic regimes significantly improved the *in vivo* muscle performance of A/J mice (Table 1) but did not have a significant effect on either *ex vivo* muscle performance or the presence of centrally-nucleated myofibers (supplementary material Table S1), suggesting the presence of continued regeneration in AchE.I-treated A/J mice. Taken together, these findings show that treatment of A/J mice with the AchE.I Pyridostigmine bromide prevents the CMAP decrement and substantially improves the *in vivo* muscle performance of muscles lacking Dysferlin.

## Discussion

While Dysferlin is expressed in many cell types, muscle expression is sufficient to rescue disease pathophysiology in mice (Millay et al., 2009). Until recently, it was largely thought that this muscle-specific role was related to the membrane repair activity of Dysferlin at the muscle plasma membrane, since a major pathological hallmark of Dysferlinopathies is membrane damage with an accumulation of sub-sarcolemmal membrane vesicles (Glover and Brown, 2007; Han and Campbell, 2007). However, a recent study showed that even when a minimal Dysferlin expression construct sufficient to restore membrane repair in isolated muscle fibers was transgenically expressed in mice, muscle damage and weakness continued to occur *in vivo* (Lostal et al., 2012). One possible explanation for this surprising result is that sarcolemmal repair *in vivo* differs from repair in isolated fibers. Another possibility is that Dysferlin, in addition to its role in membrane repair, is also involved in other physiological processes in muscle and that loss of these functions plays important roles in disease pathogenesis. Outside of its role in muscle membrane repair, other physiological roles for Dysferlin in muscle have not been described. Our discovery that loss of Dysferlin affects cholinergic signaling in both worms and mice could represent one such additional role for Dysferlin in muscle.

Our studies show that old, but not young, Dysferlin mutant mice exhibit frequency-dependent RNS EMG defects. While these phenotypes are consistent with the progressive nature of disease onset in humans, they could also result from the ageing process itself. Indeed, previous work shows that ageing does cause progressive synaptic degeneration at morphological and functional levels (Balice-Gordon, 1997; Li et al., 2011). However, significant morphological changes occur primarily after 24 months of age and most functional alterations in synaptic activity occur within the first 6 weeks of age. Given that our studies were carried out during a period where there are minimal reported age-related changes in muscle structure/function (2 months to 15 months of age), it seems unlikely that our data are simply due to physiological consequences of ageing. In support of this, we found that when A/HeJ mice, the control strain for the A/J mouse Dysferlin model (http://jaxmice.jax.org), were aged in parallel to the A/J mince, they did not exhibit significant RNS EMG rundown or alterations in *ex vivo* contractile properties (Table 1; supplementary material Table S1).

We have interpreted our findings in a way that suggests the RNS EMG defect associated with loss of Dysferlin is not related to chronological age but rather is related to a specific role for Dysferlin in the regulation of cholinergic signaling. Regardless of the specific mechanism(s), we find that treatment with the AchE.I pyridostigmine can rescue the functional declines in muscle performance in Dysferlin mutants. Interestingly, histopathological properties of the disease, such as the presence of centrally nucleated fibers, were unaffected, perhaps suggesting that cholinergic defects are a consequence rather than the underlying cause of disease. Nevertheless, this is a highly significant and translational finding as there are currently no established treatments for LGMD2B. Whether pyridostigmine treatment rescues muscle functional performance in Dysferlin mutant mice because it enhances cholinergic signaling or because it prevents age-induced declines in cholinergic signaling remains an open question that warrants future study.

While our data show a small but statistically significant and reproducible decrease in the high-frequency RNS CMAP response of Dysferlin mutant mice (∼5% at 3 Hz in A/J), the applicability of these findings to humans is currently unknown. Currently, there is no evidence for NMJ functional defects in LGMD2B patients and standard EMGs in these patients is reportedly normal. However, standard clinical EMGs do not always utilize a RNS protocol and whether or not LGMD2B patients exhibit RNS-evoked defects remains an open question that warrants more clinical study in light of our findings. While human patients with defects in cholinergic signaling, such as those with Myasthenia gravis, can exhibit substantial RNS rundowns of 20% or more, some bonafide Myasthenia gravis patients exhibit reduced or even no RNS rundown (Stalberg, 1980; Claussen et al., 1995), suggesting that even small EMG defects can be reflective of underlying NMJ dysfunction. Further study is needed to determine if the small synaptic defects we observe in Dysferlin mutant mice represents an atypical presentation of synaptic dysfunction, whether these defects are present only in a subset of muscle groups, and how relevant synaptic dysfunction may be to disease pathogenesis in humans.

While our findings show that Dysferlin contributes to muscle cholinergic signaling in both worms and mice, we have not defined the molecular or cell biological mechanism(s) through which Dysferlin acts or whether Dysferlin exerts such action via its localization at the NMJ or other subcellular location. Given its role as a regulator of membrane fusion (Han and Campbell, 2007), one hypothesis is that Dysferlin controls synaptic AchR levels via regulated insertion/retrieval of AchR-containing vesicles. However, our studies in worms suggest that steady-state synaptic AchR levels are not altered (supplementary material Fig. S2). Whether Dysferlin coordinates activity-dependent AchR turnover is an open question that will require more sophisticated live-animal imaging approaches.

Aside from regulating vesicular insertion of AchRs at the synapse, Dysferlin could act in additional ways to promote cholinergic signaling. For example, Dysferlin could regulate the activity of synaptic AchRs without altering AchR abundance or turnover. Dysferlin could also act outside of the synapse to regulate cholinergic signaling via effects on excitation–contraction coupling or regulation of sarcoplasmic Ca^2+^ levels (Roche et al., 2011). Regardless of the mechanism, our finding that treatment with the synapse-specific AchE.I pyridostigmine restores appropriate synaptic function and rescues muscle strength in Dysferlin mutant mice suggests that elevating synaptic Ach levels can compensate for this defect. While these pharmacological findings support the hypothesis that there is a deficit of Ach signaling in Dysferlin mutant mice, they also suggest the intriguing possibility that LGMD2B patients might benefit from AchE.I therapy. Given the long clinical history and general long-term safety of these compounds (Maggi and Mantegazza, 2011), more pre-clinical studies to examine the potential benefit of AchE.I therapy for LGMD2B patients are warranted. In this regard, it is important to note that in our studies, AchE.I therapy improved muscle physiology, but did not affect other markers of disease (i.e. CNFs). Therefore, it seems likely that, at best, a combined therapeutic approach targeting both AchR synaptic dysfunction and defective membrane repair processes may be needed to best address the sequalae of LGMD2B.

## Materials and Methods

### Ethics statement

All animals were handled in strict accordance with good animal practice as defined by the relevant national and/or local animal welfare bodies, and all animal work was approved by the appropriate committee: Institutional Animal Care and Use Committee (IACUC), University of Pennsylvania Perelman School of Medicine, Philadelphia, PA 19104 (Protocol no. 802799).

### Nematode culture

*C. elegans* strains were maintained at 20°C under standard conditions (Brenner, 1974). The wild-type strain was Bristol N2; mutations used include: *fer-1(hc1)*, *fer-1(hc24)*, *fer-1(b232)*, *fer-1(hc47)*, *unc-49(e409)*, *unc-119(ed3)*. The molecular alterations caused by the various *fer-1* mutations are as follows (positions based on fer-1a isoforms); hc1 – G290E, hc24 – L1809F, b232 – S1486N, hc47 – W494STOP. Since the *fer-1* alleles are temperature sensitive, animals used for assays were synchronized by the hypochlorite method then grown at the restrictive temperature of 25°C to generate staged young adults.

### Transgenes for cell-specific *fer-1* expression

Cell-specific expression constructs were created using the Gateway cloning strategy (Invitrogen) and each contained a cell-specific promoter, the *fer-1* genomic sequence, and the *unc-54* 3′ untranslated region. The *myo-3* promoter (muscle-specific), *spe-11* promoter (sperm-specific) and *unc-119* promoter (neuron-specific) transgenes *drSi6*, *drSi16*, and *drSi19*, respectively, were chromosomally integrated into the chromosome II ttTi5605 Mos site using single copy insertion (Frøkjær-Jensen et al., 2008). Germline transformation of the *fer-1p::gfp::unc-54* 3′ UTR PCR fusion product and *him-4p::Mb::yfp* plasmid was performed using standard techniques (Mello et al., 1991).

### Fluorescence microscopy

*C. elegans* were immobilized with 3 mM levamisole (Sigma) on 3% agar pads. Fluorescence Z-stack images were obtained using a Leica DMI4000B microscope (63× objective) with a Leica DFC340Fx digital camera. The width of muscle arms from ventral left body wall muscle 11 was measured at the midpoint of each arm (Leica advanced fluorescence 6000 software).

For the UNC-63::YFP experiments, *fer-1* mutants were crossed into the *unc-63::YFP* strain (Gendrel et al., 2009) using standard genetic methods. All strains were verified to be homozygous for the *unc-63::yfp* marker by PCR genotyping. Fluorescence Z-stack images of the neuromuscular junctions were acquired as described above. All analyses were carried out on collapsed Z-stacks of the raw images and quantified in Leica Advanced Fluorescence 6000 software.

### Pharmacological assays

Assay plates were prepared by adding aldicarb (Fisher) or levamisole (Sigma) stock solutions to NGM agar at 50°C to a final concentration of 1 mM (aldicarb) or 0.5 mM (levamisole). After pouring, plates were stored at 4°C and used within one week. For each assay, 30 animals/genotype were picked to drug plates (10 animals/plate) and prodded every 15 min. (aldicarb assay) or 10 min. (levamisole assay). Worms that failed to respond were classified as paralyzed. All experiments were conducted by two independent researchers who were blinded to genotype and repeated at least two times.

### Mice

Male A/HeJ and A/J, mice were purchased from Jackson Laboratories. Two groups (*n* = 10) of A/J mice were treated with Pyridostigmine bromide (Sigma–Aldrich) dissolved in water (0.32 mg/ml water) available *ad libitum*. The treatment for one group began at the age of 8 weeks (pre-disease onset) and at the age of 7 months for the second group (co-disease onset) and continued until the age of 14 months for both groups. Age matched control groups received no treatment.

### *In vivo* repetitive nerve stimulation protocol

Mice were anesthetized (Tribromoethanol 0.4–0.75 mg/kg, intraperitoneal injection) and placed in a prone position. A 2.0 cm incision was made exposing the biceps femoris muscle, and the artery *genus descendes* was used as a landmark (Bourquin et al., 2006). The sciatic nerve was isolated by blunt dissection. The tibial and sural nerves were cut, leaving the common peroneal nerve intact. Following nerve preparation, the tissue was hydrated with saline solution and mouse body temperature maintained using a heat lamp.

A specially designed apparatus was constructed to simultaneously record the muscle compound action potential (CMAP) and muscle contractile force following nerve stimulation, based on previous reports (Ashton-Miller et al., 1992; Gorselink et al., 2001). The apparatus was constructed of acetal polymer and consisted of a lower platform, measuring 30 cm × 25 cm, and a secondary platform, measuring 20 cm × 10 cm which was raised approximately 1.5 cm off the lower platform. The mouse was supine on the lower platform with the right leg positioned on the raised platform. A force transducer (FT03, Grass Technologies) coupled to a foot plate was positioned on the raised platform and the foot was secured to the footplate using 6.0 nylon suture. A micromanipulator was used to position stimulation electrodes around the sciatic nerve. Additional micromanipulators were used to position needle recording electrodes into the tibialis anterior muscle, which allowed the acquisition of CMAPs. The force transducer allowed the acquisition of muscle contractile forces resulting from dorsiflexion of the anterior compartment of the lower leg at the ankle joint. Data were acquired using an A/D converter and accompanying software (LabChart V6, ADInstruments) (supplementary material Fig. S3). CMAP and force features were quantified using custom MATLAB scripts, which were validated through comparisons with manually compiled data.

A low to high frequency repeated stimulation protocol was performed, consisting of a set of 10 contractions at the following stimulation frequencies: 0.1, 0.5, 1.0, 3.0, and 10 Hz. Stimulations lasted 0.1 ms at a voltage of 5 V. One minute rest was allowed in between each set, and this protocol was repeated three times to ensure that the synaptic decrement was not due to degeneration of the preparation. In all cases, A/J and SJL/J mice showed normal responses to low frequency stimulation (0.1 Hz) after exhibiting CMAP decrement at high frequency (3 or 10 Hz) stimulation, indicating that the defect was due to the physiological properties of the muscle and not to the loss of integrity of either the preparation or the recording setup. *Ex vivo* EDL muscle contractile properties were examined in freshly dissected EDL muscles, as previously described (Pistilli et al., 2011; Bogdanovich et al., 2002). Upon completion of the *in vivo* experiments, mice were euthanized by placing them in a 100% CO_2_ chamber for three minutes, followed by cervical dislocation.

### Muscle histology

Muscles were imbedded in Tissue Freezing Medium (TBS, Durham, NC), flash frozen, and stored at −80°C. Frozen sections (10 µm thick) of quadriceps muscle were obtained using a cryostat at maintained at −21°C and placed onto glass slides (Superfrost/Plus, Fisher Scientific). Sections were fixed in ice-cold methanol for 5 min and then processed for histological examination by hematoxylin and eosin-phloxine (H&E) staining. Digital images were acquired using an Olympus BX51 microscope at 40×. The number of centrally nucleated fibers was determined by manual inspection.

### Statistics

All data are presented as means ± S.D., unless noted otherwise. Data were analyzed using either the Students T-test for comparisons between two groups or one-way ANOVA analysis with Boneferroni's correction for comparisons between 3 or more groups. For the levamisole and aldicarb resistance assays, the number of motile animals over time was analyzed using the Logrank test as implemented in Graphpad Prism 4. *P* values of <0.05 were taken to indicate statistical significance.

## Supplementary Material

Supplementary Material
