# Supplementary material for: FER-1/Dysferlin promotes cholinergic signaling at the neuromuscular junction in C. elegans and mice
Source: Biol Open. 2013 Oct 15;2(11):1245–52. doi: 10.1242/bio.20135637 (PMC3828772; doi:10.1242/bio.20135637)
Supplement: Supplementary Material [file supp_2_11_1245__index.html]

FER-1/Dysferlin promotes cholinergic signaling at the neuromuscular junction in C. elegans and mice — Supplementary Material 

# FER-1/Dysferlin promotes cholinergic signaling at the neuromuscular junction in *C. elegans* and mice

## bio.20135637 Supplementary Material

**Files in this Data Supplement:**

- Supplementary Material - Predrag Krajacic et al. doi: 10.1242/bio.20135637
