# Supplementary material for: FER-1/Dysferlin promotes cholinergic signaling at the neuromuscular junction in C. elegans and mice
Source: Biol Open. 2013 Oct 15;2(11):1245–52. doi: 10.1242/bio.20135637 (PMC3828772; doi:10.1242/bio.20135637)
Supplement: Supplementary Material [file supp_bio.20135637_bio.20135637-s1.pdf]

Biology Open

Supplementary Material

Predrag Krajacic et al. doi: 10.1242/bio.20135637

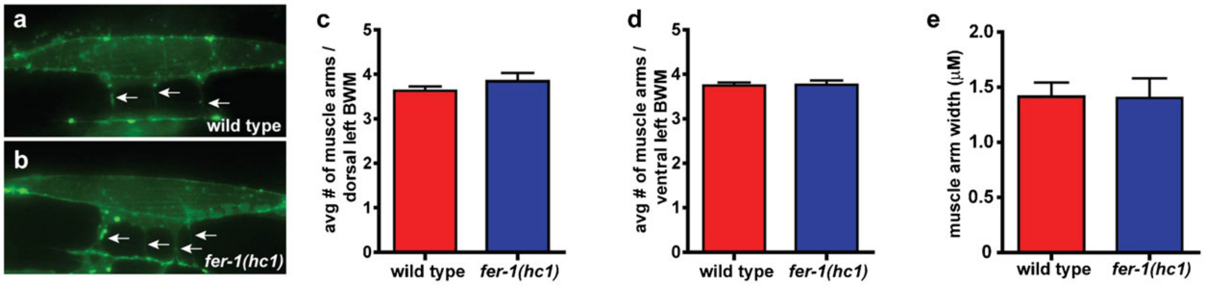

Fig. S1. Loss of *fer-1* does not disrupt body-wall muscle arm number or morphology. (a,b) Representative images of ventral left quadrant body-wall muscle 11 from wild-type (a) and *fer-1(hc1)* mutant (b) animals, visualized with membrane-anchored YFP to show muscle arms (arrows). (c,d) No significant difference in the number of muscle arms from body-wall muscles in the dorsal left (c, $P=0.34$) or ventral left (d, $P=0.89$) quadrant was observed between wild-type and *fer-1(hc1)* mutant animals. $n>30$ muscles/genotype for the dorsal left quadrant and $n>80$ muscles/genotype for the ventral left quadrant. (e) No significant difference in the width of muscle arms from the ventral left body-wall muscle 11 was observed between wild-type and *fer-1(hc1)* mutant animals; $n\geq44$ muscle arms measured per genotype, $P=0.96$. For (c–e), error bars indicate SE; P values were determined using Student's t-test.

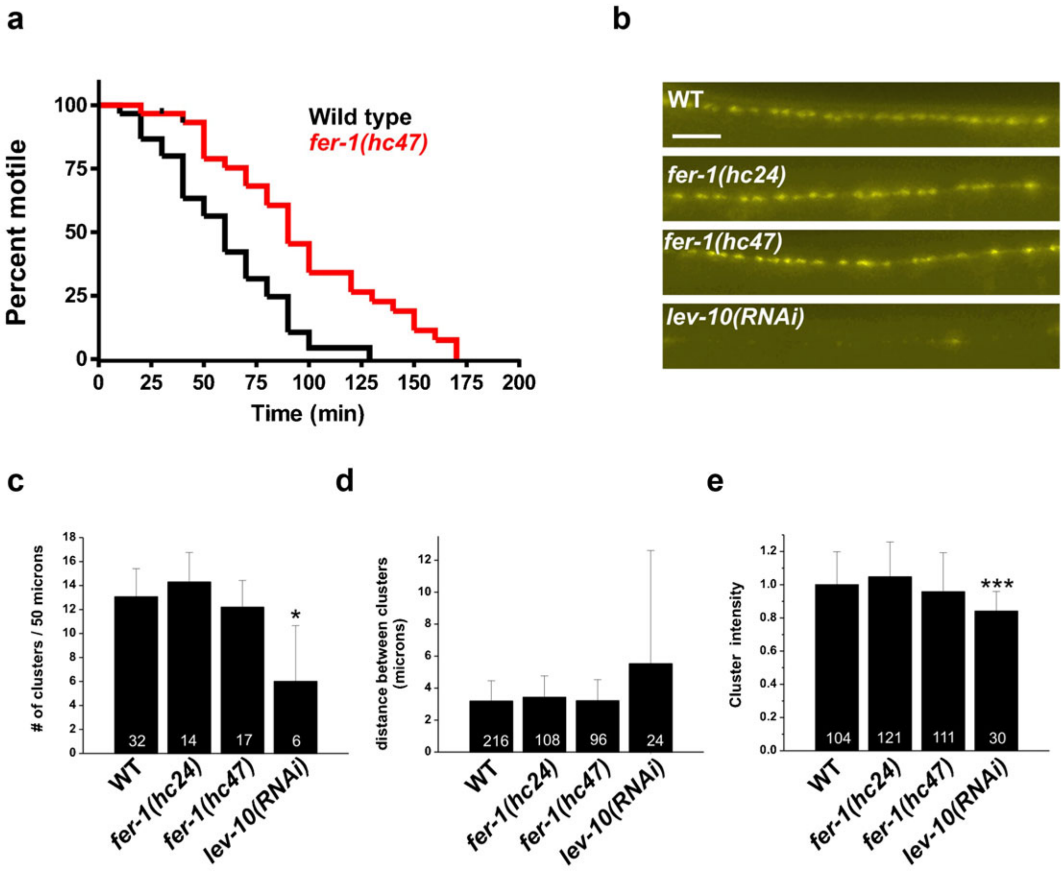

Fig. S2. Loss of *fer-1* does not disrupt the clustering or abundance of the L-AchR subunit UNC-63 at the *C. elegans* neuromuscular junction. (a) Levamisole resistance in the *fer-1* null mutant *hc47*. Logrank test $P<0.001$. (b) Representative wide field fluorescence images of a functional UNC-63::YFP fusion protein (Gendrel et al., 2009) in the indicated genetic backgrounds. Scale bar=10 µm. (c–e) Quantification of the density (c), spacing (d), and abundance (e) of UNC-63::YFP clusters at the neuromuscular junction in the indicated genetic backgrounds. *lev-10(RNAi)*, which disrupts L-AchR clustering (Gally et al., 2004) was used as a positive control. Data shown are mean ± S.D. $^{***}P<0.001$, $^{*}P<0.05$, non-parametric one-way ANOVA with Dunn's Multiple Comparison post-hoc testing. The numbers within the bars indicate the 'N' for each measurement. There were no statistically significant differences between either of the *fer-1* mutants and wild type for any of these properties.

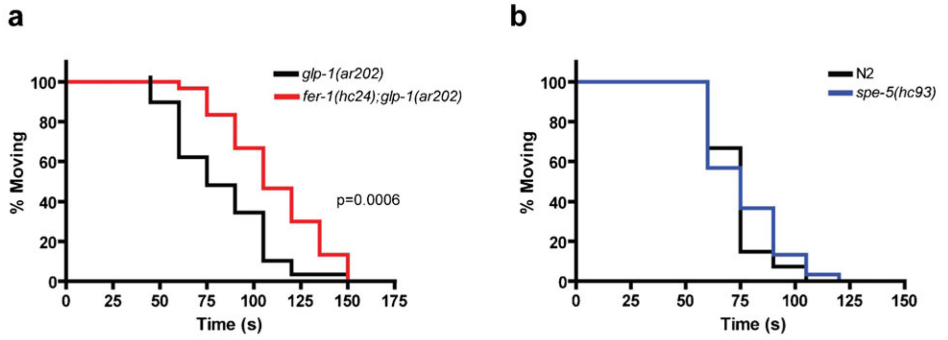

Fig. S3. *fer-1*-dependent reductions in cholinergic signaling do not depend on *fer-1* germline function and are not phenocopied by other sperm defective mutants. (a) *fer-1* aldicarb resistance in the *glp-1(ar202)* mutant background; n=30/strain, Logrank test $P<0.001$. (b) Spermatogenesis defective mutant *spe-5(hc93)* does not exhibit levamisole resistance. (n=30/strain, Logrank test $P>0.05$).

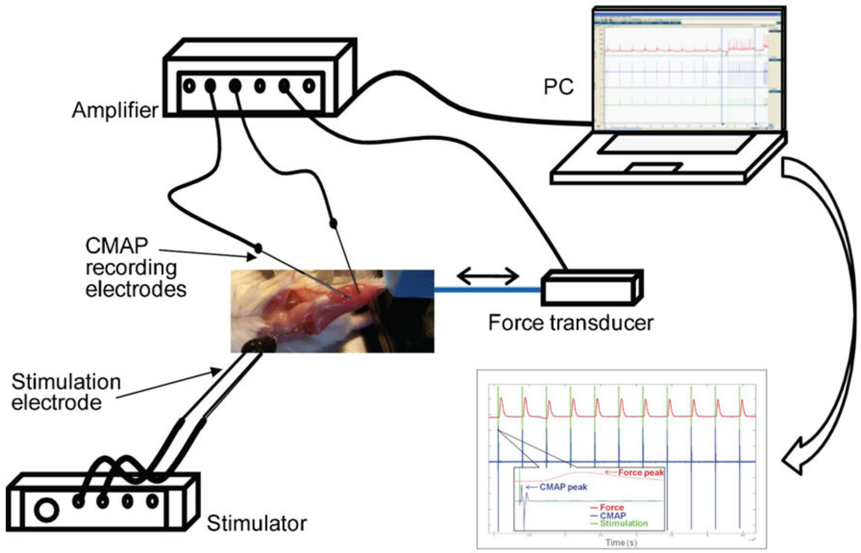

Fig. S4. *In vivo* repetitive nerve stimulation platform setup. Simultaneous recording of the muscle compound action potential (CMAP) and the resulting muscle contractile force following repetitive nerve stimulation. CMAPs were obtained by needle recording electrodes inserted into the tibialis anterior muscle.

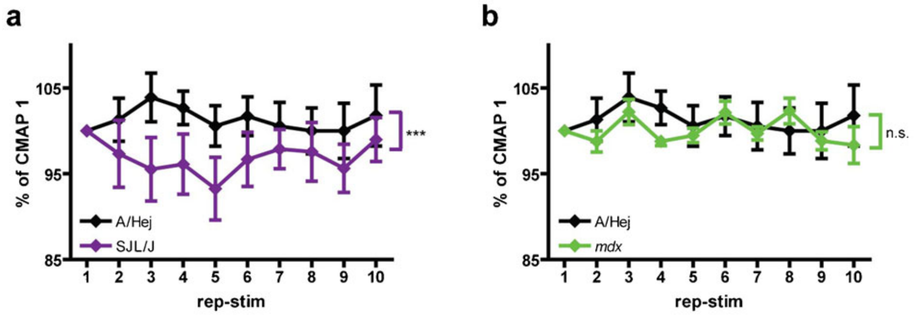

Fig. S5. CMAP decrement observed in another dysferlin mutant (SJL/J), but not in a non dysferlin muscular dystrophy mouse model (*mdx*). (a,b) Normalized mean CMAP voltage on 3 Hz repetitive nerve stimulation, expressed as a % of CMAP 1 (a) CMAP decrement in dysferlin deficient SJL/J mice (n=8). 1 way ANOVA with Bonferroni's multiple comparison test: ***$P<0.001$. (b) Dystrophin deficient *mdx* mice (n=5). 1 way ANOVA with Bonferroni's multiple comparison test, $P>0.05$.

Table S1. *Ex vivo* contractile properties of EDL muscles.

Mouse Strain	A/HeJ[1] (*n*=12)	A/J[1] (*n*=11)	A/J[1,2] (post-onset Rx) (*n*=16)	A/J[1,2] (pre-onset Rx) (*n*=12)	A/J[3] (young) (*n*=10)	SJL/J[4] (*n*=11)
EDL Wt (mg)	9.5 ± 0.81	9.2 ± 1.3	11.1 ± 1.6*	11.2 ± 0.78*	10.2 ± 0.89	13.1 ± 1.6***
EDL Lo (mm)	12.0 ± 0.63	11.3 ± 1.2	11.9 ± 0.98	11.2 ± 0.63	11.1 ± 0.65	12.1 ± 0.66
EDL CSA (mm^2)	1.7 ± 0.12	1.7 ± 0.27	1.9 ± 0.33	2.1 ± 0.11***	1.9 ± 0.18	2.3 ± 0.24***
Twitch (mN)	97.1 ± 26.5	66.4 ± 20.9*	93.4 ± 25.1	92.2 ± 23.6	124.5 ± 23.4	87.9 ± 15.4
Twitch/CSA (mN/mm^2)	58.4 ± 15.5	38.5 ± 11.6	50.6 ± 21.4	44.4 ± 12.5	65.7 ± 12.5	39.0 ± 8.5*
Tetanus (mN)	414.1 ± 122.5	289.2 ± 56.6	420.3 ± 113.5	459.8 ± 79.6	503.0 ± 99.5	440.2 ± 108.6
Tetanus/CSA (mN/mm^2)	248.6 ± 73.3	172.7 ± 62.6	217.6 ± 83.1	221.2 ± 46.7	265.1 ± 46.7	195.0 ± 55.8
ECC (%)	17.7 ± 10.2	10.4 ± 8.8	12.6 ± 6.5	19.2 ± 9.6	11.0 ± 7.9	14.1 ± 7.1
Twitch/Tetanus	0.24 ± 0.06	0.23 ± 0.07	0.23 ± 0.07	0.20 ± 0.04	0.25 ± 0.05	0.22 ± 0.04
CNF (%)	1.4 ± 0.17	32.5 ± 5.2***	46.3 ± 12.3***	n.d.	n.d.	n.d.

[1]age=14.5 months
[2]treated with 0.32 mg/ml Pyridostigmine bromide
[3]age=2 months
[4]age=10 months
Data presented as Mean ± S.D., and analyzed using one-way ANOVA with Bonferroni's post-hoc test. The null hypothesis was rejected when $P<0.05$.
Wt=muscle weight, Lo=muscle optimal length, CSA=cross sectional area, CNF=centrally nucleated fiber, n.d.=not determined
*$P<0.05$; **$P<0.001$; ***$P<0.0001$

Biology Open
